# Supplementary material for: Risk of retinal detachment and exposure to fluoroquinolones, common antibiotics, and febrile illness using a self-controlled case series study design: Retrospective analyses of three large healthcare databases in the US
Source: PLoS One. 2022 Oct 6;17(10):e0275796. doi: 10.1371/journal.pone.0275796 (PMC9536641; doi:10.1371/journal.pone.0275796)
Supplement: S1 File — (PDF) [file pone.0275796.s001.pdf]

Janssen Research & Development*

Study Protocol for Retrospective Observational Studies Using Secondary Data

A Self-Controlled Case Series Study of Fluoroquinolones Exposure and Collagen-related Serious Adverse Events

Protocol RRA-19796

* Janssen Research & Development (Janssen R&D) is a global organization that operates through different legal entities in various countries. Therefore, the legal entity acting as the sponsor for studies of Janssen R&D may vary. The term "sponsor" is used throughout the protocol to represent these various legal entities.

Status: Approved
Date: 25 July 2017
Prepared by: Janssen Research & Development, LLC
EDMS number: EDMS-ERI-145948739, 1.0

Confidentiality Statement
The information in this document contains trade secrets and commercial information that are privileged or confidential and may not be disclosed unless such disclosure is required by applicable law or regulations. In any event, persons to whom the information is disclosed must be informed that the information is *privileged* or *confidential* and may not be further disclosed by them. These restrictions on disclosure will apply equally to *all* future information supplied to you that is indicated as *privileged or confidential.*

TABLE OF CONTENTS

TABLE OF CONTENTS **2**

LIST OF ABBREVIATIONS **3**

1. RESPONSIBLE PARTIES **4**
1.1. Investigators and Authors 4
1.2. Sponsor 4

2. ABSTRACT **4**

3. AMENDMENT AND UPDATES **5**

4. RATIONALE AND BACKGROUND **6**

5. STUDY OBJECTIVES **9**
5.1. Research Question 9
5.2. Objectives 9

6. RESEARCH METHODS **9**
6.1. Study Design and Setting 9
6.2. Data Source(s) 9
6.3. Study Population 10
6.4. Outcome(s) of Interest: Definition of Event. 11
6.5. Exposure(s) of Interest 12
6.6. Other Variables of Interest 13

7. SAMPLE SIZE AND STUDY POWER **13**

8. DATA ANALYSIS **14**
8.1. Calculation of Incidence Rate Ratio 14
8.2. Patient Characteristics Summary 16
8.3. Sensitivity Analyses 16

9. STRENTHS AND LIMITATIONS OF THE RESEARCH METHODS **17**

10. PROTECTION OF HUMAN SUBJECTS **18**

11. MANAGEMENT AND REPORTING OF ADVERSE EVENTS AND ADVERSE REACTIONS **18**

12. PLANS FOR DISSEMINATING AND COMMUNICATING STUDY RESULTS **18**

13. REFERENCES **18**

LIST OF ABBREVIATIONS

**Abbreviation**	**Definition**
FQ	Fluoroquinolone
SCCS	Self-controlled case series
RD	Retinal detachment
ATR	Achilles Tendon Rupture
AAD	Aortic Aneurysm and Dissection
IRR	Incidence rate ratio
CI	Confidence interval

1. RESPONSIBLE PARTIES

1.1. Investigators and Authors

Role	Name; Title; Affiliation
Primary Operating Company Point	Chantal Holy, PhD
Project Lead	Chantal Holy, PhD
Clinical and Design Input	Daniel Fife, MD
Clinical Expert	Angelina Villasis Keever, MD
Primary Programmer/Analyst	James Weaver; Ajit Londhe
Epidemiologist/QC Analyst	Jill Hardin, PhD
External consultant	N/A
Add more rows as needed	

1.2. Sponsor

Sergio Fonseca
Sr Director Portfolio CDTL
Established Products
1125 Trenton-Harbourton Rd
Titusville NJ 08560

2. ABSTRACT

Title: A Self-Controlled Case Series Study of Fluoroquinolones Exposure and Collagen-related Serious Adverse Events

Rationale and background: Studies with divergent findings were recently published on the potential association of exposure to oral fluoroquinolones (FQ) and risk of certain adverse events related to collagen [1-4]. This study is therefore designed to address in a self-controlled case series (SCCS) whether association of Achilles Tendon rupture (ATR), retinal detachment (RD) or aortic aneurysm and dissection (AAD) with FQ was observed and whether it was different from that of other exposures (other antibiotic or febrile illness). In this study, ATR, RD and AAD are evaluated separately as 3 distinct endpoints. The other antibiotics or febrile conditions evaluated herein will help define potential confounding by indication. The use of the SCCS design protects against potential confounding by personal characteristics such as obesity and smoking that vary little over 2-year periods and are poorly captured by most health care databases. This study should therefore help evaluate whether FQ has an association with collagen-related adverse events that may be different from that observed with other antibiotics or febrile illness, in a study design that controls for individual confounders.

Research question and objectives:

Research question: Using an SCCS study design, determine the association between FQ exposure as well as other exposures (amoxicillin, azithromycin, trimethoprim (without sulfamethoxazole) and trimethroprim/sulfamethoxazole, and febrile illness

not treated with antibiotics) and ATR, RD, and AAD. (The endpoints: ATR, RD and AAD are analyzed separately.) The SCCS study design controls for confounding by factors such as obesity and smoking, as all patients serve as their own controls.

Primary objective: Same as above

Data sources: Truven CCAE and Medicare (Supplemental) and Optum ClinFormatics (Optum)

Study period: The study period will be April 1st, 2011 to March 31st, 2017 for all observations, but from April 1st, 2012 to March 31st, 2017 for all outcomes.

Study design: Self-controlled case series using retrospective claims database.

Population: All patients with ATR or RD or AAD (aka "Event"), and exposures to FQ or any of the other antibiotics noted above or febrile illness not treated with antibiotic, within a defined study period and at least 1 year of continuous enrollment prior to the event.

Data Analysis: Incidence rate ratios (IRR) and 95% confidence intervals (CI) will be calculated from conditional Poisson regression, which measure the potential associations between each risk factor (FQ, other antibiotics and febrile condition) and either ATR, RD or AAD. Analyses will be conducted using R.

3. AMENDMENT AND UPDATES

Table 1: Amendments and Updates

Number	Date	Section of study protocol	Amendment or update	Reason
1	3/29/2017	Throughout the protocol: clarifications	Clarifications of existing text	PREP review
2	10/9/2017	6.3: Study Population<br><br>6.3 Estimates<br><br>6.3 Exclusion criteria: Inherited disorders<br><br>8.3 Sensitivity Analyses	Increased duration of study to most recent available data (March 2017)<br><br>Estimates to be presented for each database and as pooled estimates<br><br>Clarification: these disorders are exclusion criteria for RD as well as ATR and AAD.<br><br>Clarification added to the definition of population for RD and AAD in the sensitivity analyses.	Inclusion of most recent available data<br><br>Clarification<br><br>Clarification<br><br>Clarification
3	4/19/2018	6.3 Exclusion Criteria	Clarification: patients with events occurring during the at-risk period of more than one exposure are excluded.	Clarification – eliminate cases where events cannot be associated with one exposure

		8.1 Calculation of incidence risk ratio	Clarification: include text related to standard analytical approaches. In this case, including the analyses of pre-exposure incidence risk ratios.	Clarification
4	8/1/2018	5.1 Research Question<br><br>6.3 Study Population<br><br><br><br>8.1 Calculation of Incidence Rate Ratio	Clarification: Trimethoprim, when written by itself, refers to trimethoprim without sulfamethoxazole<br><br>Clarification: pooled estimate to be provided for all outcomes<br><br>Clarification: no events in pre-index period<br><br>Clarification: p value calibration – impact on findings	Clarification<br><br>Clarification<br><br>Clarification<br><br>Clarification
5	8/1/2018	8.3 Sensitivity Analyses	Amendment: addition of a post-hoc sensitivity analysis as Section 8.3.2	Evaluate the possible effect of patients being hospitalized prior to events and given medications during those hospitalizations. These medications would not be captured in the databases.
6	6/12/2019	8.3 Sensitivity Analyses	Amendment: addition of a post-hoc sensitivity analysis as Section 8.3.3	Exclude potential impact of antibiotic ophthalmic drops and ophthalmic topical preparation formulations from analyses related to retinal detachment (RD). Re-run all RD analysis *excluding* these formulations from the antibiotic definitions.

4. RATIONALE AND BACKGROUND

Several recent studies have associated exposure to oral fluoroquinolones (FQs) with an increased risk of serious adverse events related to collagen: ATR or other tendon injuries[4], RD [5 6] (though a meta-analysis found no association [7]), and AAD [2 3]. Two studies of the association of FQs with an increased risk of ATR found an increase within 60 days of exposure [4 8] and a third found it within 90 days [1]. Of the two studies that found an association of FQs with an increased risk of aortic aneurism and aortic dissection, one found the increased risk in the 60 days of exposure[3], the other in the 30 days following exposure [2]. The first study that observed an association of FQs with an increased risk of RD found the increased risk during the current use [5].

Because FQs are prescribed for infections, the observed association may reflect an effect of infections rather than an effect of FQs, i.e. the observation may be confounded by indication (infection). If so, we would expect to see that the association is not specific to FQs, i.e. that other antibiotics and perhaps even febrile illness not treated with antibiotics are also associated with the above adverse events. Several of the studies cited above have examined the possibility of association of their endpoints with other antibiotics. One study of FQs and ATR found an association with trimethoprim/sulfonamides [8] but no association with other antibiotics. Another study found a weak association with exposure to FQs and a similar weak association with exposure to azithromycin, but no association with exposure to clarithromycin [1]. A study of FQs and all three of the above endpoints also found an association between exposure to amoxicillin and each of those endpoints [2], but found that this association was weaker than the association with FQs for the aortic aneurism outcome and the Achilles tendon rupture outcome. The initial study of FQs and RD also examined the association with beta-lactam antibiotics and found none [5].

The issue of confounding by indication is therefore still unresolved when assessing FQ, even though regulatory interpretation of the data seems to indicate no association.[1]

To address this question, further analyses are required.

In addition, a key limitation of prior studies is the fact that many confounders that are known to affect collagen-based outcomes related to comorbidities or life choices are not consistently documented in databases. For example, for AAD, risk factors include aspects such as height, blood pressure, smoking status as well as heredity – information typically missing from any observational database [9 10]. To reduce the impact of such unidentifiable confounders on study outcomes, a self-controlled case series (SCCS) study design will be used in this study.

The self-controlled case series (SCCS) was first described by Farrington *et al* in the context of vaccine research and has since been used for other safety evaluations [11]. This approach is based on the idea that each subject acts as its own control. The period of time during which subjects are being evaluated is defined as the observation period. During the observation period, subjects may be exposed to experimental agents and for each exposure, time-at-risk is defined as the time period after exposure during which risk for the event of interest is increased.

The main advantage of the SCCS approach is it allows inferences about relative incidence of adverse events during risk periods of exposures versus unexposed control periods using cases only – each subject serving as its own control. Typical subject-to-

[1] https://www.fda.gov/safety/medwatch/safetyinformation/safetyalertsforhumanmedicalproducts/ucm513065.htm, accessed 5/26/2017.

subject variability and confounding by factors such as obesity and smoking are therefore inherently controlled. Not controlled however may be changes in subject presentation that may occur over time, over the duration of the observation period. For this reason, observation periods may be kept relatively short and age adjustments are made.

Key assumptions of the SCCS design are as follows: 1) events must be rare or recurrence must be independent. All events considered here (ATR, RD or AAD) are rare events; 2) There needs to be variation of subjects' age or variation of exposure status with time of event. This assumption is met in this study; 3) Exposure time is not limited by events. This assumption is also met in this study. A final assumption of the traditional SCCS requires exposures to be independent of events. [12] This assumption is not met in this study as occurrence of a ATR, RD or AAD may deter physicians from prescribing FQ. to account for this limitation, a recently-described modified method of SCCS that adjusts for studies where occurrence of events affects post-event exposures will be used herein. [13]

The outcomes of interest in this study relate to adverse events resulting from collagen-related disorders, specifically ATR, RD and AAD. These conditions will be analyzed separately using 3 distinct cohorts, one for each condition. There will not be any composite outcome.

Both ATR and RD are acute events that require immediate medical attention. It can therefore be hypothesized that there is very little time difference between the time stamp of AT rupture or RD identification in the database vs. that of the condition's actual inception. In addition, prior studies have proposed methodologies to identify AT rupture and RD in databases using algorithms that combine diagnostic and procedural codes [1 14]. For these two conditions, it is possible to assume that one can identify the conditions and their approximate start date in observational databases.

Most aneurysms go undetected for long periods of time and grow slowly over many years. Studying whether or not there is an association between FQ and AAD is therefore complicated by the fact that there is no ascertaining of the exact date at which the aneurysm really formed. Lee *et al* conducted their study using the date of the first diagnosis of aortic aneurysm or aortic dissection as inception date and evaluating whether these events were linked to use of FQ either concurrently (i.e., prescription filled within 60 days prior to event) or previously (i.e., within days 61 to 365 prior to event date). Lee *et al* suggested an association in the current use time window. [3]

5. STUDY OBJECTIVES

5.1. Research Question

Determine the association between FQ exposure as well as other exposures (amoxicillin, azithromycin, trimethoprim (without sulfamethoxazole)[2] and trimethroprim/sulfamethoxazole, and febrile illness not treated with antibiotics) and ATR, RD, and AAD.

Distinct results will be generated for FQ exposure and each other exposure.

5.2. Objectives

Evaluate whether there is an increased risk of ATR, RD or AAD following exposure to FQ or other antibiotics (amoxicillin, azithromycin, trimethoprim and trimethroprim/sulfamethoxazole) and febrile illness not treated with antibiotics, using a study design that minimizes the impact of patient-specific unidentifiable confounders such as heredity and smoking.

6. RESEARCH METHODS

6.1. Study Design and Setting

A SCCS study design will be used for this analysis. Patients will be defined as having continuous enrollment for at least 1 year and have at least one instance of ATR, RD or AAD as defined below, as well as at least 1 exposure as described in the Exposures of Interest section. Inclusion and exclusion criteria are further defined below.

6.2. Data Source(s)

The study will be replicated in 3 databases (Truven CCAE and Medicare (Supplemental) and Optum ClinFormatics (Optum)) as recent work suggests that results from retrospective studies may vary by database [15]).

The Truven Health MarketScan® Research Databases (CCAE and Medicare) provide longitudinal healthcare utilization information through claims databases for more than 133 million patients, detailed at the patient level, and includes inpatient, outpatient and prescription claims. The Commercial database (CCAE) include more than 133 million patients under 65 and with employer-sponsored insurance, whereas the Truven Medicare database includes 9.76 million patients with supplemental Medicare insurance.

The Optum Clinformatics database comprises administrative claims data submitted for payment by providers and pharmacies that are verified, adjudicated, adjusted and de-identified. This database also includes tables with inpatient confinement, lab

[2] All mentions of trimethoprim by itself in this document refers to trimethoprim without sulfamethoxazole.

results and member eligibility data. Overall, 77.4 million patients are included in the database.

Whereas the databases are largely separate, there is no assurance against overlap (i.e., patients represented in both databases). This issue is also identified in limitations section.

6.3. Study Population

(Note: This being a SCCS, the cases and controls are the same – there is therefore no sub-heading to account for cases vs controls.)

All codes related to conditions and prescriptions mentioned herein are listed in a separate code list document.

Patients will be included in the study if they have evidence of at least one instance of ATR, RD or AAD, as defined below, and at least 1 exposure as described in the Exposures of Interest section. Achieving one of the endpoints (ATR, RD or AAD) at any time ends the eligibility for achieving another endpoint (i.e., one patient may only have one of these 3 endpoints, the first occurring in the medical history). The study period will be April 1st 2012 to March 31st, 2017 (a prior study having evaluated RD prior to April 1st 2012) [14]).

All subjects will also have at least one (1) year of continuous enrollment with pharmacy benefits prior to the ATR, RD or AAD. This period of time will be required to ensure subjects did not have collagen-related conditions that may have caused the ATR, RD or AAD and may begin a year prior to the study period (April 1st 2011 to April 1st 2012).

The observation time for each patient will include the time they are observed in the database within the study period. Each patient's observation period will end at the end of the study period or when their observation time in the database ends. A limiting assumption of the standard SCCS method is that both exposure distribution and observation period be independent of time of event (in our case, event being ATR, RD or AAD) [12]). This assumption is violated in our study since the likelihood of exposure is likely reduced after the index; however, to account for such study designs, the analytical method developed by Farrington *et al* will be applied [13]. This method is applicable for examining a temporal association between multiple exposures and a "terminal" event such as ATR, RD or AAD.

The ATR, RD or AAD event is described below as the *index* event.

The associations between each exposure and ATR, RD or AAD will be estimated in each database separately as well as presented as estimates pooled across study databases using a random-effect analysis model as described by Dersimonian & Laird [16].

Exclusion criteria

Subjects will be excluded from the study if they meet any of the following exclusion criteria:

- For ATR, AAD and RD cohorts:
  - Subjects experience the index event while within a time-at-risk window for more than one exposure type. (The exposure types in this study include FQ as a class, amoxicillin, azithromycin, trimethoprim, trimethroprim/sulfamethoxazole, and febrile illness not treated with antibiotics. Events while within a time-at-risk window of two or more exposure types cannot be associated with any one exposure. Patients are therefore excluded from the study).
  - Subjects have an ATR/AAD or RD in the 1-year pre-index period
- For ATR and AAD cohort:
  - Subjects have inherited disorders of connective tissue, specifically: Ehlers-Danlos syndrome, epidermolysis bullosa, Marfan syndrome, osteogenesis imperfecta
  - Subjects have a secondary ATR repair procedure any time before and up to 7 days after the index ATR diagnosis.
- For RD cohort:
  - Subjects have inherited disorders of connective tissue, specifically: Ehlers-Danlos syndrome, epidermolysis bullosa, Marfan syndrome, osteogenesis imperfecta
  - Subjects have cataract surgery prior to index
  - Subjects have iridotomy or iridectomy prior to index
  - Subjects have a diagnosis of eye injury during the period prior to the event
  - Subjects have endophthalmitis or retinitis during the observation period

6.4. Outcome(s) of Interest: Definition of Event.

In this study, each outcome (ATR, RD, and AAD) are analyzed separately.

Achilles Tendon Rupture: Daneman *et al*, Wise *et al* and Van der Linden *et al* found associations between AT rupture and FQ using a 30-day window following a treatment course, citing among others a post-market surveillance report that suggests onset of tendinopathy at around 18 days (±24.5 days) [2 4 17 18]. However, evidence from spontaneous reports may be swayed towards earlier time frames because events that occur soon after an exposure are more likely to be reported than those occurring later. Additional data points include: Daneman *et al*, who reported a median time from FQ initiation to tendon rupture of 19 days [2]. Van der Linden in 2003 suggested odds ratio for AT rupture of 4.3 (95% CI: 2.4-7.8), 2.4 (95% CI: 1.5-3.7) and 1.4 (95%CI: 0.9-2.1) within 1, 6 and 18 months of exposures, respectively.[8] For this study, the time-at-risk window will therefore be 30-day + the days of treatment, such that total time window will include days of treatments with an additional 30 days. The sensitivity analyses listed below will evaluate a 2-month risk period, thus addressing the increased OR reported by van der Linden for the 2-month time frame.

Subjects will be defined as having ATR if they receive a diagnosis for Achilles tendon rupture as well as one of the following procedures: tenotomy or primary ruptured AT repair (with or without graft) within 7 days of diagnosis. Index will be based on the earlier date of diagnosis or procedure.

Retinal Detachment: Association between FQ and RD were reported by Etminan looking at the current use of FQ, current being defined as having an index date overlapping with the end of a prescription date. [5] Other studies have suggested associations beyond the prescription date, including Kuo *et al*, who identified onset of RD at 35.5 days post use of FQ. [6] Daneman *et al* reported only marginal association between FQ and RD, using however a 30-day window. All these data points suggest that the time-at-risk window is early after use of FQ and for this study, a similar time window as for AT rupture will be applied, i.e. the total time window will include days of treatments with an additional 30 days, and a sensitivity analysis will increase this at-risk period to time of treatment + 60 days.

Subjects will be defined as having a RD if they received a diagnosis of RD and a procedure for RD, e.g.: sclera buckle, vitrectomy, retinopexy, retinal cryotherapy, silicone oil fill, air gas fluid exchange or pneumatic retinopexy (see annex for full list), within 14 days of index, as described by Etminan *et al* [5] and Fife *et al* [14]. Index will be defined as the earlier date of diagnosis or procedure.

Aortic Aneurysm and Dissection: Daneman *et al* and Lee *et al* both reported associations between FQ exposure and increased risk for AAD. Daneman's risk period was limited to 30 days after a treatment course. Lee *et al* observed the greatest association in the 60 days following prescription. [2 3] To ensure consistency with prior studies, our analysis will include a 30-day period following last day of prescription, such that the entire risk period will include treatment days with an additional 30 days, and a sensitivity analysis will increase this period to 60 days.

Subjects will be defined as having AAD if they received a primary diagnosis for aortic aneurysm, aortic rupture or dissection and have also received an aortic repair surgical procedure concurrently to the AAD diagnosis, in an inpatient or emergency department setting. Index will be defined as the earlier date of diagnosis or procedure.

Please see associated excel file, sheet "Outcomes" for listing of outcomes and associated definitions and concepts.

Please see associated excel file, sheet "analysis specifications" for listing of analyses of exposures, outcome and time risk (days).

6.5. Exposure(s) of Interest

The FQ evaluated herein include all oral forms of FQ (24 hours extended release tablets, extended release tablets, oral solution, oral suspension, oral table, pack). The

drugs included are ciprofloxacin, gatifloxacin, gemifloxacin, levofloxacin, moxifloxacin, norfloxacin, ofloxacin – lumped as a class.

Other antibiotics to analyze include amoxicillin, azithromycin, trimethoprim and trimethroprim/sulfamethoxazole. Febrile illness not treated with antibiotics will also be analyzed as an exposure. Each exposure will be analyzed separately.

Febrile illness not treated with antibiotics: Febrile illness not treated with antibiotics is defined in this study as:

1) concurrent diagnoses of viral disease (ICD-9: V73.99) *with* concurrent fever (ICD-9:780.60), and no concurrent prescription for any antibiotic during the 60-day period before and after the first date of viral disease diagnosis.

or

2) A diagnosis of influenza (ICD-9: 487) with no concurrent inpatient admission during the 60-day period pre- or post-influenza diagnosis, and no prescription for any antibiotics during the 60-day period pre- or post-influenza diagnosis.

In addition to the exposures noted above, a list of exposures known to have no association with the outcomes will be used as negative controls. The complete list of negative controls is included in the attached code list.

Please see associated excel file, sheet "Exposures" for listing of exposures and associated definitions and concepts for exposures.

6.6. Other Variables of Interest

The following additional variables will be collected for all patients.

- Age
- Drug use, including steroids (Injected vs oral)

(Due to the self-controlled nature of the study, usual patient variables such as sex, CCI connective tissue disease, obesity and diabetes are not included in this list – only time varying confounders are listed herein.)

7. SAMPLE SIZE AND STUDY POWER

As indicated in prior publications evaluating RD, calculations of the minimum detectable relative risk (α=0.05 and power = 0.80) suggests that each database has sufficient sample to identify relative risks ≥ 1.1 for any exposure to ciprofloxacin or levofloxacin and relative risks ≥ 2.0 for moxifloxacin, gatiflxacin, gemifloxacin, ofloxacin and norfloxacin [14]. In our study, all FQs are treated as a class and the minimum detectable relative risk was calculated using previously described methods. [19] The results in Table 2 below and confirm that across all databases there is sufficient sample size to identify relative risks as listed below for FQ as a class, amoxicillin, azithromycin, trimethoprim and trimethoprim/sulfamethoxazole, for each of the outcomes described above (RD, AAD and ATR).

Table 2: Minimum detectable relative risk for each outcome and exposure.

Outcome	Exposure	Minimum Detectable Relative Risk
RD	FQ exposure + 30 days	1.2807
RD	Amoxicillin exposure + 30 days: Amoxicillin	1.3596
RD	Azithromycin exposure + 30 days: Azithromycin	1.4465
RD	Trimethoprim exposure + 30 days: Trimethoprim	1.4394
RD	Trimethoprim/sulfamethoxazole exposure + 30 days	1.4394
RD	FQ exposure + 60 days	1.225
RD	Amoxicillin exposure + 60 days: Amoxicillin	1.2761
RD	Azithromycin exposure + 60 days: Azithromycin	1.3304
RD	Trimethoprim exposure + 60 days: Trimethoprim	1.3584
RD	Trimethoprim/sulfamethoxazole exposure + 60 days	1.3584
AAD	FQ exposure + 30 days	1.1233
AAD	Amoxicillin exposure + 30 days: Amoxicillin	1.1156
AAD	Azithromycin exposure + 30 days: Azithromycin	1.1566
AAD	Trimethoprim exposure + 30 days: Trimethoprim	1.1954
AAD	Trimethoprim/sulfamethoxazole exposure + 30 days	1.1953
AAD	FQ exposure + 60 days	1.0976
AAD	Amoxicillin exposure + 60 days: Amoxicillin	1.0905
AAD	Azithromycin exposure + 60 days: Azithromycin	1.1189
AAD	Trimethoprim exposure + 60 days: Trimethoprim	1.1582
AAD	Trimethoprim/sulfamethoxazole exposure + 60 days	1.1581
ATR	FQ exposure + 30 days	1.7075
ATR	Amoxicillin exposure + 30 days: Amoxicillin	1.6238
ATR	Azithromycin exposure + 30 days: Azithromycin	1.7179
ATR	Trimethoprim exposure + 30 days: Trimethoprim	1.7725
ATR	Trimethoprim/sulfamethoxazole exposure + 30 days	1.7715
ATR	FQ exposure + 60 days	1.5481
ATR	Amoxicillin exposure + 60 days: Amoxicillin	1.4774
ATR	Azithromycin exposure + 60 days: Azithromycin	1.5301
ATR	Trimethoprim exposure + 60 days: Trimethoprim	1.6219
ATR	Trimethoprim/sulfamethoxazole exposure + 60 days	1.621

8. DATA ANALYSIS

8.1. Calculation of Incidence Rate Ratio

Incidence rate ratio (IRR) and 95% confidence intervals (CI) will be calculated to quantify the relative risk of RD, ATR, and AAD between periods of exposure and non-exposure to FQ and each of the other exposures listed above.

The exposure distribution is likely altered at time of the outcome because prescribing physicians are likely aware of the suspected adverse effects of FQs, which violates the model assumption that event times are independent from the exposure distribution and

observation period. To address this non-independence, time periods after events will be analyzed using methods developed by Farrington *et al* and described elsewhere. [13]

To further evaluate any potential confounding by indication, the *pre*-exposure relative incidence of events for each exposure (using periods occurring 60 to 30 days and 29 to 1 day prior to index) and each event will be estimated. These analyses will be conducted using standard analytical practice as described elsewhere.[20]

Exposure and outcome rate can vary with age, which can lead to confounding. Age effects can be accounted for by splitting the observation period into age-specific intervals, the lengths of which are used as weights in conjunction with exposed and unexposed times to derive the Poisson incidence rate for the observation period as a whole. [12]

Exposures to other drugs may represent time varying confounders that warrant statistical correction. For example, non-steroidal anti-inflammatory drugs may be prescribed to treat connective tissue disorders. Because the association of interest is that of FQs on RD/ATR/AAD, this effect can be separated from that of other drugs through statistical adjustment. Regularized multiple SCCS will be executed to account for the potentially confounding effects of all other drug exposures.[21] Since most drug exposures are not associated with RD/ATR/AAD, the parameter estimates of most other drug exposures will be zero. Drug exposures additionally can serve as a proxy for time-varying conditions that may be under treatment.

Empirically calibrated p-values will be generated to account for residual bias that may be inherent to the study design. Whereas traditional p-value calculations account for random error, calibrated p-values reflect the probability of observing an effect estimate under an empirically derived null hypothesis that accounts for both random and systematic error. Instead of using the theoretical null value of $\log(\beta)=0$ against which statistical hypothesis tests are made, a null distribution of actual effects on negative control exposures on study outcomes will be generated and used in its place. If systemic error is inherent in the study design, then the empirical null distribution will be $\log(\beta)!=0$. P-values are subsequently calculated against the null distribution.[22]

The results from the calibrated p-value will be used to determine the suitability of the estimates. Severe systematic bias may require excessively large/small estimate and that may otherwise be inconclusive. Moderate or small systematic bias will require suitably large/small estimates, which may be appropriate for dissemination.

Analyses will be conducted separately for each outcome (RD/ATR/AAD) using R packages SelfControlledCaseSeries and EmpiricalCalibration.

Results from each database will be pooled using a random-effect analysis model as described by Dersimonian & Laird.[16]

8.2. Patient Characteristics Summary

Descriptive statistics will be generated for all patients in the study. A separate table will be generated for ATR, RD and AAD. Each table will show age (mean and standard deviation), percentage female, observation duration after index and in total (median (IQR) – expressed in years).

For each event (ATR, RD and AAD), a separate table will be generated indicating the number of events (No, %) by type of exposure. The FQ will be shown as a consolidated line item as well as broken down by FQ type. (FQ type: Ciprofloxacin, levofloxacin, norfloxacin, moxifloxacin, gatifloxagin, gemifloxacin, ofloxacin). All other exposures will be shown as a consolidated line item as well as broken down by antibiotic (amoxicillin, azithromycin, trimethoprim and trimethroprim/sulfamethoxazole) and febrile illness, as described above.

8.3. Sensitivity Analyses

8.3.1 The following sensitivity analyses will be performed:

- For analysis of RD: the sensitivity analysis will be like the main analysis except that subjects will require prior ophthalmologist visit at any time prior to the RD to further ensure no prior RD and access to specialty ophthalmology care.
- For AAD: because aneurysms may be detected incidentally during admissions, the sensitivity analysis will be performed on patients given an aortic diagnostic imaging procedure followed within 30 days with an AAD diagnosis. (Diagnostic imaging will need to happen prior to the diagnosis to exclude cases where an imaging procedure was conducted simply to rule out AAD. Unlike the other AAD cohort, this cohort may or may not have received an aortic aneurysm repair procedure. For these patients, the index date will be the date of the diagnostic imaging procedure).
- For all 3 outcomes: a sensitivity analysis will be conducted assuming a 2-month time-at-risk window.

8.3.2 A post-hoc sensitivity analysis will be performed to evaluate the possible effect of patients being hospitalized prior to events and given medications during those hospitalizations. Medication provided during inpatient hospital stays are not captured in the databases; a patient receiving, for example, a FQ during an inpatient hospital stay will not have any claim in the databases associated with the use of that medication during that hospitalization.

The purpose of this post-hoc sensitivity analysis is therefore to repeat analyses performed above, excluding patients with a hospitalization within 30- and 60-days prior to AAD. (This analysis focuses on AAD because: 1) the study identified severe systemic bias for ATR, rendering any findings related to ATR inconclusive and thus eliminating the need for further sensitivity analyses for ATR, and 2) the number of patients with inpatient admissions in the 30- and 60-days before RD was less than 1% of the entire RD cohort, thus conducting the post-hoc sensitivity analysis described above for RD may not affect findings).

- For AAD: two new cohorts will be generated:
  - Cohort 1: The first cohort will include all the patients as defined in this protocol *except* those patients with an inpatient hospitalization with a discharge date within 30 days prior to AAD.

- Cohort 2: The second cohort will include all the patients as defined in this protocol *except* those patients with an inpatient hospitalization with a discharge date within 60 days prior to AAD.

All the analyses outlined in this protocol, as well as the sensitivity analyses listed above in Section 8.3.1, will be conducted on Cohort 1 and Cohort 2 (separately).

Please see excel file sheet "Analysis Specifications" for complete list of analysis to be conducted (inclusive of sensitivity analyses listed above).

8.3.3 A post-hoc sensitivity analysis will be conducted on RD events as following: all primary and sensitivity analyses will be re-run for all exposures of interest, as described in this protocol. Specifically, the analyses to be conducted are:

1. Primary Outcomes, risk window: 30 days + exposure period
2. Sensitivity Outcomes, risk window: 60 days + exposure period
3. Pre-Exposure Windows with Primary Outcomes, risk window: 30 days + exposure period
4. Other Drugs with Primary Outcomes, risk window: 30 days + exposure period

However, for all antibiotic exposures (FQ class, amoxicillin, azithromycin, trimethoprim and trimethroprim with sulfamethoxazole, trimethroprim without sulfamethoxazole), only drugs in the following formulations will be included: tablets (oral and chewable), extended-release oral tablets, pack. Formulations such as ophthalmic solution and solutions/ointments will be excluded because they do not produce a systemic effect and may likely be prescribed in association with an ophthalmic eye condition or procedure.

9. STRENTHS AND LIMITATIONS OF THE RESEARCH METHODS

- The SCCS study design controls for confounders not typically described in the databases, as explained above
- Confounding by indication has already been suggested for several antibiotics in multiple studies, and this study may therefore have an ambiguous finding. [1]
- In addition, the study has all the usual limitations of retrospective database analyses, including impact of coding errors or missing subject information. Usual database limitations also include the inability to measure non-coded/reported confounders. (This limitation is lessened by the SCCS study design.)
- For AAD: Onset of disease may happen long before aneurysms are diagnosed. Because of that, this study identifies the time of presentation of ADD rather than the time of onset. Outcomes are also limited to the most extreme form of identification (as primary diagnoses during inpatient/emergency room visits).
- Statistical adjustments are being made for patients who die shortly after an event, or because of an outcome (e.g. aneurysm).
- Outcomes calculated using series of negative controls will provide greater credibility to the findings.
- Please see excel file sheets "Neg controls RD", "Neg controls AAD", and "Neg controls ATR" for listing of negative control medications for outcome.
- Whereas the databases reflect mostly separate populations, there is no assurance against overlap, i.e. same patients captured in separate databases.

10. PROTECTION OF HUMAN SUBJECTS

The use of Optum, CCAE and MDCR was reviewed by the New England Institutional Review Board and was determined to be exempt from broad Institutional Review Board approval as this project did not involve human subject research. [3]

11. MANAGEMENT AND REPORTING OF ADVERSE EVENTS AND ADVERSE REACTIONS

The study will be registered into TMS. Adverse events will be reported according to the company's Standard Operating Procedures

12. PLANS FOR DISSEMINATING AND COMMUNICATING STUDY RESULTS

A final report will be created from study results which will be submitted for publication in a peer reviewed journal as outlined in the Johnson & Johnson Publication Policy.

13. REFERENCES

1. Seeger JD, West WA, Fife D, et al. Achilles tendon rupture and its association with fluoroquinolone antibiotics and other potential risk factors in a managed care population. Pharmacoepidemiology and drug safety 2006;**15**(11):784-92.
2. Daneman N, Lu H, Redelmeier DA. Fluoroquinolones and collagen associated severe adverse events: a longitudinal cohort study. BMJ open 2015;**5**(11):e010077.
3. Lee CC, Lee MT, Chen YS, et al. Risk of Aortic Dissection and Aortic Aneurysm in Patients Taking Oral Fluoroquinolone. JAMA internal medicine 2015;**175**(11):1839-47.
4. van der Linden PD, Sturkenboom MC, Herings RM, et al. Fluoroquinolones and risk of Achilles tendon disorders: case-control study. Bmj 2002;**324**(7349):1306-7.
5. Etminan M, Forooghian F, Brophy JM, et al. Oral fluoroquinolones and the risk of retinal detachment. Jama 2012;**307**(13):1414-9.
6. Kuo SC, Chen YT, Lee YT, et al. Association between recent use of fluoroquinolones and rhegmatogenous retinal detachment: a population-based cohort study. Clinical infectious diseases : an official publication of the Infectious Diseases Society of America 2014;**58**(2):197-203.
7. Alves C, Penedones A, Mendes D, et al. A systematic review and meta-analysis of the association between systemic fluoroquinolones and retinal detachment. Acta ophthalmologica 2016;**94**(5):e251-9.
8. van der Linden PD, Sturkenboom MC, Herings RM, et al. Increased risk of achilles tendon rupture with quinolone antibacterial use, especially in elderly patients taking oral corticosteroids. Archives of internal medicine 2003;**163**(15):1801-7.

[3] Nasim NG. Optum Clinformatics Data Mart (CDM) data dictionary. Optum; 2012.

9. Smelser DT, Tromp G, Elmore JR, et al. Population risk factor estimates for abdominal aortic aneurysm from electronic medical records: a case control study. BMC cardiovascular disorders 2014;**14**:174.
10. Kent KC, Zwolak RM, Egorova NN, et al. Analysis of risk factors for abdominal aortic aneurysm in a cohort of more than 3 million individuals. Journal of vascular surgery 2010;**52**(3):539-48.
11. Farrington CP, Nash J, Miller E. Case series analysis of adverse reactions to vaccines: a comparative evaluation. American journal of epidemiology 1996;**143**(11):1165-73.
12. Whitaker HJ, Farrington CP, Spiessens B, et al. Tutorial in biostatistics: the self-controlled case series method. Statistics in medicine 2006;**25**(10):1768-97.
13. Farrington CP, Whitaker HJ, Hocine MN. Case series analysis for censored, perturbed, or curtailed post-event exposures. Biostatistics 2009;**10**(1):3-16.
14. Fife D, Zhu V, Voss E, et al. Exposure to oral fluoroquinolones and the risk of retinal detachment: retrospective analyses of two large healthcare databases. Drug safety 2014;**37**(3):171-82.
15. Madigan D, Ryan PB, Schuemie M, et al. Evaluating the impact of database heterogeneity on observational study results. American journal of epidemiology 2013;**178**(4):645-51.
16. DerSimonian R, Laird N. Meta-analysis in clinical trials. Controlled clinical trials 1986;**7**(3):177-88.
17. Wise BL, Peloquin C, Choi H, et al. Impact of age, sex, obesity, and steroid use on quinolone-associated tendon disorders. The American journal of medicine 2012;**125**(12):1228 e23-28 e28.
18. Khaliq Y, Zhanel GG. Fluoroquinolone-associated tendinopathy: a critical review of the literature. Clinical infectious diseases : an official publication of the Infectious Diseases Society of America 2003;**36**(11):1404-10.
19. Musonda P, Farrington CP, Whitaker HJ. Sample sizes for self-controlled case series studies. Statistics in medicine 2006;**25**(15):2618-31.
20. Petersen I, Douglas I, Whitaker H. Self controlled case series methods: an alternative to standard epidemiological study designs. Bmj 2016;**354**:i4515.
21. Simpson SE, Madigan D, Zorych I, et al. Multiple self-controlled case series for large-scale longitudinal observational databases. Biometrics 2013;**69**(4):893-902.
22. Schuemie MJ, Gini R, Coloma PM, et al. Replication of the OMOP experiment in Europe: evaluating methods for risk identification in electronic health record databases. Drug safety 2013;**36 Suppl 1**:S159-69.

Appendix: Table list

1) **Patient Demographics**
   Table for each exposure listed in the protocol.
   (Table 1.1: ATR, Table 1.2: RD, Table 1.3 AAD - Each table having the same information as below:
   - Total Sample size
   - Age (mean, standard deviation)
   - % Female
   - Observation Period (mean, median)
   - Exposed Time (for each exposures)
   - Time pre-event (mean, median)
   - Time post-event (mean, median)
   - Year of Index)

2) **Outcomes tables: All outcomes tables are listed in a separate excel document**
