# Supplementary material for: Risk of retinal detachment and exposure to fluoroquinolones, common antibiotics, and febrile illness using a self-controlled case series study design: Retrospective analyses of three large healthcare databases in the US
Source: PLoS One. 2022 Oct 6;17(10):e0275796. doi: 10.1371/journal.pone.0275796 (PMC9536641; doi:10.1371/journal.pone.0275796)
Supplement: S2 File — Negative Control Diagnostics and Incidence Rate Ratio Estimates for Retinal Detachment in all Databases, for Primary, Sensitivity and Post-hoc Analyses. (RTF) [file pone.0275796.s002.rtf]

Supporting Information


Figure S1: Distribution of effect estimates for RD following negative control exposures. Each blue dot represents the estimated incidence rate ratio (x axis) and standard error (y axis) of each negative control exposure. Estimates below the dashed line have uncalibrated p < 0.05. Estimates in the orange area have calibrated p < 0.05. The red band indicated the 95% credible interval around the boundary of the orange area. 
OPTUMEXTDOD	IBMMDCR	IBMCOM	
			


Table S1: IRR estimates for RD in OPTUMEXTDOD, from the primary analysis (antibiotic exposures include all antibiotic formulations). The risk window is defined as the exposure period plus 30 days.
Exposure	IRR (60d to 30d)	95% CI LB (60d to 30d)	95% CI UB (60d to 30d)	IRR (29d to 1d)	95% CI LB (-29d to 1d)	95% CI UB (29d to 1d)	IRR	95% CI LB	95% CI UB	p	Calibrated p	
FQ class	1.404	1.251	1.570	9.591	9.037	10.175	4.138	3.876	4.414	0.000	0.000	
FINTA	0.739	0.262	1.623	0.902	0.354	1.868	1.020	0.457	1.951	0.957	0.917	
Amoxicillin	0.876	0.748	1.018	0.636	0.529	0.758	0.696	0.600	0.801	0.000	0.069	
Azithromycin	0.843	0.700	1.006	0.623	0.501	0.765	0.741	0.621	0.875	0.001	0.138	
Trimethoprim without Sulfamethoxazole	1.822	1.211	2.629	34.433	29.823	39.767	11.127	9.491	13.032	0.000	0.000	
Trimethoprim with Sulfamethoxazole	0.674	0.463	0.945	0.611	0.413	0.868	0.655	0.485	0.866	0.004	0.074	
Key: IRR = Incidence rate ratio, CI = Confidence Interval, LB = Lower Bound, UB = Upper Bound, p = p-value, Calibrated p = Empirically Calibrated p-value, FINTA = febrile illness not treated with antibiotics	


Table S2: IRR estimates for RD in OPTUMEXTDOD, from the post-hoc sensitivity analysis (antibiotic exposures include tablets only). The risk window is defined as the exposure period plus 30 days.
Exposure	IRR (60d to 30d)	95% CI LB (60d to 30d)	95% CI UB (60d to 30d)	IRR (29d to 1d)	95% CI LB (-29d to 1d)	95% CI UB (29d to 1d)	IRR	95% CI LB	95% CI UB	p	Calibrated p	
FQ class	1.015	0.857	1.193	0.727	0.595	0.878	0.753	0.639	0.882	0.001	0.147	
FINTA	0.722	0.256	1.584	0.885	0.347	1.830	1.264	0.621	2.280	0.480	0.602	
Amoxicillin	0.808	0.646	0.997	0.704	0.552	0.883	0.759	0.626	0.911	0.004	0.167	
Azithromycin	0.865	0.721	1.028	0.684	0.556	0.831	1.037	0.893	1.198	0.624	0.837	
Trimethoprim without Sulfamethoxazole	0.000	-	-	0.000	-	-	1.772	0.300	7.714	0.490	0.527	
Trimethoprim with Sulfamethoxazole	0.664	0.456	0.931	0.569	0.380	0.816	0.778	0.588	1.009	0.067	0.248	
Key: IRR = Incidence rate ratio, CI = Confidence Interval, LB = Lower Bound, UB = Upper Bound, p = p-value, Calibrated p = Empirically Calibrated p-value, FINTA = febrile illness not treated with antibiotics	


Table S3: IRR estimates for RD in IBMMDCR, from the primary analysis (antibiotic exposures Include all antibiotic formulations). The risk window is defined as the exposure period plus 30 days.
Exposure	IRR (60d to 30d)	95% CI LB (60d to 30d)	95% CI UB (60d to 30d)	IRR (29d to 1d)	95% CI LB (-29d to -1d)	95% CI UB (-29d to -1d)	IRR	95% CI LB	95% CI UB	p	Calibrated p	
FQ class	1.367	1.142	1.624	7.672	6.938	8.475	4.354	3.929	4.819	0.000	0.000	
FINTA	1.148	0.064	5.429	1.323	0.074	6.282	0.000	-	2.116	-	-	
Amoxicillin	0.804	0.616	1.029	0.838	0.644	1.072	0.630	0.488	0.801	0.000	0.000	
Azithromycin	0.939	0.693	1.242	0.533	0.353	0.767	0.676	0.493	0.903	0.011	0.012	
Trimethoprim without Sulfamethoxazole	1.829	0.818	3.522	43.366	33.831	55.713	13.196	9.949	17.463	0.000	0.000	
Trimethoprim with Sulfamethoxazole	1.064	0.655	1.629	0.602	0.310	1.045	0.533	0.311	0.851	0.014	0.014	
Key: IRR = Incidence rate ratio, CI = Confidence Interval, LB = Lower Bound, UB = Upper Bound, p = p-value, Calibrated p = Empirically Calibrated p-value, FINTA = febrile illness not treated with antibiotics	


Table S4: IRR estimates for RD in IBMMDCR, from the post-hoc sensitivity analysis (antibiotic exposures include tablets only). The risk window is defined as the exposure period plus 30 days.

Exposure	IRR (60d to 30d)	95% CI LB (60d to 30d)	95% CI UB (60d to 30d)	IRR (29d to 1d)	95% CI LB (29d to 1d)	95% CI UB (-29d to -1d)	IRR	95% CI LB	95% CI UB	p	Calibrated p	
FQ class	0.995	0.773	1.258	0.843	0.639	1.090	0.992	0.797	1.220	0.938	0.634	
FINTA	1.101	0.062	5.193	1.273	0.071	6.025	0.000	-	2.096	-	-	
Amoxicillin	0.744	0.480	1.097	1.033	0.706	1.455	0.854	0.604	1.173	0.353	0.272	
Azithromycin	0.913	0.673	1.208	0.523	0.347	0.753	0.985	0.757	1.260	0.910	0.664	
Trimethoprim without Sulfamethoxazole	0.000	-	9.496	0.000	-	10.337	1.739	0.215	9.979	0.572	0.599	
Trimethoprim with Sulfamethoxazole	1.046	0.651	1.586	0.622	0.330	1.057	0.736	0.468	1.101	0.160	0.125	
Key: IRR = Incidence rate ratio, CI = Confidence Interval, LB = Lower Bound, UB = Upper Bound, p = p-value, Calibrated p = Empirically Calibrated p-value, FINTA = febrile illness not treated with antibiotics	


Table S5: IRR estimates for RD in IBMCOM, from the primary analysis (antibiotic exposures Include all antibiotic formulations). The risk window is defined as the exposure period plus 30 days.

Exposure	IRR (60d to 30d)	95% CI LB (60d to 30d)	95% CI UB (60d to 30d)	IRR (29d to 1d)	95% CI LB (29d to 1d)	95% CI UB (29d to 1d)	IRR	95% CI LB	95% CI UB	p	Calibrated p	
FQ class	1.536	1.388	1.697	12.000	11.414	12.613	5.481	5.193	5.784	0.000	0.000	
FINTA	0.665	0.301	1.256	0.619	0.276	1.185	0.392	0.151	0.823	0.030	0.047	
Amoxicillin	0.788	0.700	0.885	0.678	0.596	0.768	0.731	0.659	0.809	0.000	0.112	
Azithromycin	0.874	0.766	0.993	0.720	0.623	0.828	0.693	0.608	0.786	0.000	0.067	
Trimethoprim without Sulfamethoxazole	1.549	1.120	2.082	40.407	36.512	44.730	12.936	11.552	14.479	0.000	0.000	
Trimethoprim with Sulfamethoxazole	0.908	0.732	1.113	0.825	0.656	1.022	0.863	0.722	1.022	0.096	0.483	
Key: IRR = Incidence rate ratio, CI = Confidence Interval, LB = Lower Bound, UB = Upper Bound, p = p-value, Calibrated p = Empirically Calibrated p-value, FINTA = febrile illness not treated with antibiotics	


Table S6: IRR estimates for RD in IBMCOM, from the post-hoc sensitivity analysis (antibiotic exposures include tablets only). The risk window is defined as the exposure period plus 30 days.

Exposure	IRR (60d to 30d)	95% CI LB (60d to 30d)	95% CI UB (60d to 30d)	IRR (29d to 1d)	95% CI LB (29d to 1d)	95% CI UB (29d to 1d)	IRR	95% CI LB	95% CI UB	p	Calibrated p	
FQ class	0.945	0.821	1.080	0.891	0.771	1.024	0.940	0.835	1.055	0.300	0.684	
FINTA	0.669	0.303	1.264	0.616	0.276	1.178	0.453	0.189	0.907	0.048	0.065	
Amoxicillin	0.787	0.669	0.920	0.674	0.565	0.796	0.900	0.792	1.019	0.102	0.521	
Azithromycin	0.902	0.791	1.023	0.757	0.656	0.868	0.859	0.763	0.964	0.011	0.364	
Trimethoprim without Sulfamethoxazole	0.000	-	1228.480	0.000	-	1240.226	2.071	0.312	7.954	0.378	0.397	
Trimethoprim with Sulfamethoxazole	0.927	0.747	1.136	0.754	0.593	0.944	0.734	0.605	0.882	0.001	0.101	
Key: IRR = Incidence rate ratio, CI = Confidence Interval, LB = Lower Bound, UB = Upper Bound, p = p-value, Calibrated p = Empirically Calibrated p-value, FINTA = febrile illness not treated with antibiotics	


Table S7: IRR with 95% confidence intervals and calibrated p values for RD and following each exposure as indicated, for each of the databases. 
		IRR	95% CI LB	95%CI UB	C p	
FQ 	OptumExtDOD	3.030	2.844	3.227	0.000	
	IBMMDCR	3.384	3.059	3.738	0.000	
	IBMCOM	3.701	3.514	3.897	0.000	
FINTA	OptumExtDOD	1.032	0.463	1.971	0.918	
	IBMMDCR	0.000	-	2.138	-	
	IBMCOM	0.403	0.157	0.844	0.043	
Amoxicillin	OptumExtDOD	0.717	0.619	0.825	0.049	
	IBMMDCR	0.641	0.497	0.814	0.001	
	IBMCOM	0.754	0.680	0.835	0.062	
Azithromycin	OptumExtDOD	0.763	0.640	0.901	0.115	
	IBMMDCR	0.699	0.511	0.933	0.019	
	IBMCOM	0.710	0.623	0.805	0.028	
Trimethoprim without Sulfamethoxazole	OptumExtDOD	4.736	4.102	5.453	0.000	
	IBMMDCR	5.194	4.043	6.624	0.000	
	IBMCOM	5.275	4.769	5.827	0.000	
Trimethoprim with Sulfamethoxazole	OptumExtDOD	0.683	0.507	0.899	0.066	
	IBMMDCR	0.542	0.316	0.865	0.015	
	IBMCOM	0.875	0.733	1.036	0.398	
Key: IRR = Incidence Rate Ratio, CI = Confidence Interval, LB = Lower Bound, UB = Upper Bound. 
FINTA = febrile illness not treated with antibiotics


Table S8: IRR for the post-hoc analyses including tablets only, with 95% confidence intervals and calibrated p values for RD and following each exposure as indicated, for each of the databases.
		IRR	95% CI LB	95%CI UB	C p	
FQ 	OptumExtDOD	0.765	0.649	0.895	0.096	
	IBMMDCR	1.001	0.805	1.23	0.516	
	IBMCOM	0.947	0.841	1.062	0.867	
FINTA	OptumExtDOD	1.28	0.629	2.305	1.28	
	IBMMDCR	0	-	2.114	0	
	IBMCOM	0.467	0.196	0.931	0.467	
Amoxicillin	OptumExtDOD	0.775	0.639	0.93	0.775	
	IBMMDCR	0.858	0.607	1.178	0.858	
	IBMCOM	0.927	0.816	1.048	0.927	
Azithromycin	OptumExtDOD	1.064	0.916	1.228	1.064	
	IBMMDCR	1.02	0.785	1.303	1.02	
	IBMCOM	0.877	0.779	0.983	0.877	
Trimethoprim without Sulfamethoxazole	OptumExtDOD	2.012	0.35	8.588	2.012	
	IBMMDCR	1.856	0.228	10.711	1.856	
	IBMCOM	1.946	0.288	7.667	1.946	
Trimethoprim with Sulfamethoxazole	OptumExtDOD	0.809	0.613	1.047	0.809	
	IBMMDCR	0.748	0.476	1.119	0.748	
	IBMCOM	0.747	0.616	0.897	0.747	
Key: IRR = Incidence Rate Ratio, CI = Confidence Interval, LB = Lower Bound, UB = Upper Bound, 
FINTA = febrile illness not treated with antibiotics


Table S9: IRR estimates for RD in OPTUMEXTDOD from the primary analysis (antibiotic exposures include all antibiotic formulations) after adjusting for other drugs. The risk window is defined as the exposure period plus 30 days.

Exposure	IRR	95% CI LB	95% CI UB	p	Calibrated p	
FQ class	4.278	3.979	4.598	0.000	0.000	
FINTA	1.037	0.464	1.988	0.921	0.916	
Amoxicillin	0.772	0.639	0.924	0.006	0.110	
Azithromycin	0.764	0.641	0.905	0.002	0.094	
Trimethoprim without Sulfamethoxazole	6.283	5.381	7.319	0.000	0.000	
Trimethoprim with Sulfamethoxazole	0.702	0.521	0.926	0.016	0.072	
Key: IRR = Incidence rate ratio, CI = Confidence Interval, LB = Lower Bound, UB = Upper Bound, p = p-value, Calibrated p = Empirically Calibrated p-value, FINTA = febrile illness not treated with antibiotics	


Table S10: IRR estimates for RD in OPTUMEXTDOD from the post hoc analysis (antibiotic exposures include tablets only) after adjusting for exposure to other drugs. The risk window is defined as the exposure period plus 30 days.

Exposure	IRR	95% CI LB	95% CI UB	p	Calibrated p	
FQ class	0.765	0.648	0.896	0.001	0.080	
FINTA	1.288	0.632	2.326	0.446	0.595	
Amoxicillin	0.775	0.622	0.933	0.014	0.112	
Azithromycin	1.062	0.913	1.229	0.426	0.828	
Trimethoprim without Sulfamethoxazole	2.149	0.372	9.152	0.349	0.395	
Trimethoprim with Sulfamethoxazole	0.826	0.626	1.071	0.163	0.253	
Key: IRR = Incidence rate ratio, CI = Confidence Interval, LB = Lower Bound, UB = Upper Bound, p = p-value, Calibrated p = Empirically Calibrated p-value, FINTA = febrile illness not treated with antibiotics	
	


Table S11: IRR estimates for RD in IBMMDCR from the primary analysis (antibiotic exposures include all antibiotic formulations) after adjusting for exposure to other drugs. The risk window is defined as the exposure period plus 30 days.
Exposure	IRR	95% CI LB	95% CI UB	p	Calibrated p	
FQ class	4.024	3.592	4.503	0.000	0.000	
FINTA	0.000	-	2.089	-	-	
Amoxicillin	0.642	0.497	0.825	0.001	0.001	
Azithromycin	0.688	0.501	0.920	0.016	0.016	
Trimethoprim without Sulfamethoxazole	5.552	4.246	7.211	0.000	0.000	
Trimethoprim with Sulfamethoxazole	0.544	0.318	0.869	0.018	0.017	
Key: IRR = Incidence rate ratio, CI = Confidence Interval, LB = Lower Bound, UB = Upper Bound, p = p-value, Calibrated p = Empirically Calibrated p-value, FINTA = febrile illness not treated with antibiotics	
	


Table S12: IRR estimates for RD in IBMMDCR from the post hoc analysis (antibiotic exposures include tablets only) after adjusting for exposure to other drugs. The risk window is defined as the exposure period plus 30 days.
Exposure	IRR	95% CI LB	95% CI UB	p	Calibrated p	
FQ class	1.004	0.807	1.237	0.967	0.702	
FINTA	0.000	-	2.081	-	-	
Amoxicillin	0.855	0.605	1.207	0.375	0.325	
Azithromycin	1.008	0.774	1.290	0.953	0.744	
Trimethoprim without Sulfamethoxazole	1.898	0.229	11.397	0.520	0.541	
Trimethoprim with Sulfamethoxazole	0.750	0.477	1.122	0.187	0.164	
Key: IRR = Incidence rate ratio, CI = Confidence Interval, LB = Lower Bound, UB = Upper Bound, p = p-value, Calibrated p = Empirically Calibrated p-value, FINTA = febrile illness not treated with antibiotics	


Table S13: IRR estimates for RD in IBMCOM from the primary analysis (antibiotic exposures include all antibiotic formulations) after adjusting for exposure to other drugs. The risk window is defined as the exposure period plus 30 days.
Exposure	IRR	95% CI LB	95% CI UB	p	Calibrated p	
FQ class	6.435	6.052	6.839	0.000	0.000	
FINTA	0.403	0.156	0.844	0.035	0.040	
Amoxicillin	0.756	0.676	0.846	0.000	0.052	
Azithromycin	0.707	0.619	0.804	0.000	0.020	
Trimethoprim without Sulfamethoxazole	8.585	7.661	9.611	0.000	0.000	
Trimethoprim with Sulfamethoxazole	0.892	0.747	1.057	0.197	0.408	
Key: IRR = Incidence rate ratio, CI = Confidence Interval, LB = Lower Bound, UB = Upper Bound, p = p-value, Calibrated p = Empirically Calibrated p-value, FINTA = febrile illness not treated with antibiotics	
	


Table S14: IRR estimates for RD in IBMCOM from the post hoc analysis (antibiotic exposures include tablets only) after adjusting for exposure to other drugs. The risk window is defined as the exposure period plus 30 days.

Exposure	IRR	95% CI LB	95% CI UB	p	Calibrated p	
FQ class	0.965	0.855	1.085	0.557	0.657	
FINTA	0.472	0.198	0.942	0.059	0.064	
Amoxicillin	0.920	0.800	1.123	0.338	0.484	
Azithromycin	0.868	0.770	0.976	0.020	0.252	
Trimethoprim without Sulfamethoxazole	2.042	0.302	8.075	0.394	0.421	
Trimethoprim with Sulfamethoxazole	0.752	0.620	0.904	0.003	0.060	
Key: IRR = Incidence rate ratio, CI = Confidence Interval, LB = Lower Bound, UB = Upper Bound, p = p-value, Calibrated p = Empirically Calibrated p-value, FINTA = febrile illness not treated with antibiotics	
