# Supplementary material for: Risk of retinal detachment and exposure to fluoroquinolones, common antibiotics, and febrile illness using a self-controlled case series study design: Retrospective analyses of three large healthcare databases in the US
Source: PLoS One. 2022 Oct 6;17(10):e0275796. doi: 10.1371/journal.pone.0275796 (PMC9536641; doi:10.1371/journal.pone.0275796)
Supplement: Attachment — Submitted filename: Response to Reviewers.docx [file pone.0275796.s004.docx]

**RESPONSE TO THE COMMENTS: The authors would like to thank the Reviewer for the thorough comments, which have been addressed and resulted in a greatly improved manuscript.**

**COMMENTS – Manuscript PONE-D-21-26171**

Title: “Risk of Retinal Detachment and Exposure to Fluoroquinolones, Common Antibiotics, and Febrile Illness Using a Self-Controlled Case Series Study Design: Retrospective Analyses of Three Large Healthcare Databases in the US.”

**Important note: This review pertains only to ‘statistical aspects’ of the study and so** **‘clinical aspects’ [like medical importance,** **relevance of the study,** **‘clinical significance and implication(s)’ of the whole study, etc.] are to be evaluated [should be assessed] separately/independently. Further please note that any ‘statistical review’ is generally done under the assumption that (such) study specific methodological [as well as execution] issues are perfectly taken care of by the investigator(s). This review is not an exception to that and so does not cover clinical aspects {however, seldom comments are made only if those issues are intimately / scientifically related & intermingle with ‘statistical aspects’ of the study}. Agreed that ‘statistical methods’ are used as just tools here, however, they are vital part of methodology [and so should be given due importance].**

**COMMENTS:** I must say that this study is excellently conducted. Manuscript is also nicely drafted. Though it is a “pen & paper” exercise [being a ‘secondary data analyses’], hard work of the authors can be seen and is highly appreciated. In this article, I believe that, ***figures*** are very important (to understand the execution), however, in my opinion the explanation (of figures) is inadequate.

**Author response: All figure captures have been modified to include additional explanations.**

One question [sometimes] may be asked like as per line 141 of the manuscript [section on ‘Study Population’] “The study period ranged from April 1st, 2012 to March 30th, 2017”, then what are/is the reason(s) for delay in publication? is nicely answered in the same paragraph by stating that “The end-date of March 30th, 2017 was determined based on the most recent available data at the time of the analysis which ensure at least 5 years required data. {by-and-large people do not like old data analyses}

**Author response: Peer-review of our work was unfortunately delayed due to difficulties outside our control, in particular changes in focus due to COVID-19.**

Since self-controlled case series studies are rarely conducted [probably due to limitations/clumsiness] and sample size is a very important issue, little more in ‘Sample Size Assessments’ section [lines 168-70] {as well more on design - how executed} would have been appreciated.

**Author response: Additional comments were added throughout the text, to better explain our approach and findings.**

I highly appreciate a phase/sentence used in line 24 [Conclusion*:* Our results did not suggest an association between FQ and RD.] because one should always remember that “Absence of evidence is not evidence of absence” [Altman DG, Bland JM. BMJ volume 311, 1995, p 485 (Reprinted: Australian Veterinary Journal 1996;74, 311)]. {Even when P-value is not significantly lower that is null hypothesis of no difference / no association is not rejected, (in short, result is not significant), that does not amount to evidence of absence i.e., it does not imply that there no difference / no association. It only implies that there is no (i.e., these samples do not provide) [say enough] evidence to prove (rather indicate with certain specified confidence level) the difference / association}. This fact is often ignored.

**Author response: The Authors would like to thank the reviewer for reminding us of this excellent reference, which was added to our manuscript.**

Several limitations of this study as described in lines 339-355. It is true that ‘study used prescription information from claims databases’, however, note that any ‘secondary data analyses’ itself is a great limitation. I recommend acceptance of this article with few minor [these learned authors know where/what to improve] revision.

**Author response: Additional limitations were added to the discussion section.**
