# Supplementary material for: Risk of retinal detachment and exposure to fluoroquinolones, common antibiotics, and febrile illness using a self-controlled case series study design: Retrospective analyses of three large healthcare databases in the US
Source: PLoS One. 2022 Oct 6;17(10):e0275796. doi: 10.1371/journal.pone.0275796 (PMC9536641; doi:10.1371/journal.pone.0275796)
Supplement: Attachment — Submitted filename: Response to the Reviewers FINAL 2.docx [file pone.0275796.s005.docx]

**Response to Reviewers**

Reviewer #2: The authors should be commended on their methodologically rigorous approach and well written interpretation of analyses.

**Authors Response: Thank you.**

I have several comments:
1) Given the unique nature of the SCCS design, I would suggest including several more summary statistics in Table 1 relevant to this design and for each database
i) mean(sd)/median(iqr) of exposures per observation period
ii) mean(sd)/median(iqr) of intervals per observation period
iii) mean(sd)/median(iqr) of interval durations (days)
iv) total duration of observation period (days)
v) ratio of total duration of exposure/non-exposure periods (CHECK CSR)

**Author Response: The requested statistics would require considerable re-work and re-analyses, for which we are not resourced. We believe, however, that these requests might be driven by the Reviewer’s interest in the possibility that our negative findings might be due to insufficient power. Prior to the study conduct, we conducted a power calculation, as shown in the *Protocol on page 13 section 7: and reported in Sample size and Study Power (Supplemental Files)*. From our final results, the best estimate of the variability is thus given by the confidence intervals, specified for all exposures.**

2) Did the authors ensure that there was at least a 60 day observation period after the final exposure day prior to ending the observation period?

**Author Response: We thank the Reviewer for this question and have added the corresponding detail in the Methods section: all cases had a complete risk period, defined as an exposure period with a buffer of 30- or 60- days (primary vs sensitivity analyses). (*Methods: Line 148*)**

3) The risk of violating the independence assumption between RD and antibiotic exposure (not relevant for FINTA) may be significant, especially for those patients who experienced an RD event in the pre-exposure (-60 to 0 days) period for 2 reasons: i) a RD in the pre-exposure period would intuitively reduce the risk of a similar event in the post-exposure period as a patient would likely have only half the risk (one eye) as opposed to 2 eyes which might be affected by the exposure, and ii) the odds of prescribing a FQ compared to another antibiotic in those with an RD even in the pre-exposure period might be significantly reduced due to pre-existing concerns. While the authors included the pre-exposure IRR as covariates in the final regression analyses, it would be useful to conduct a sensitivity analyses to test for this violation using the following:
1) estimate the odds ratio of prescribing FQ versus other antibiotic exposure in those with pre-exposure RD event versus those without, and
2) estimate the marginal difference in the mean interval length (days) (and 95% CI) between RD-antibiotic exposure events versus antibiotic-antibiotic exposure events

**Author Response:**

- **In response to i): This is an important point. As indicated on line 111: only the first RD event was included in the study, and all patients had at least 365 days of naïve period. No patients had RD captured in the database prior to the observation period. A statement was added in the Discussion section to further reinforce this point. (*Line 354-357*).**
- **In response to ii): The Authors agree with this concern, and we further strengthened the Discussion on this topic. Cases of RD within risk periods of more than one exposure were not included. An important exclusion criterion for our study (line 156) is:**

***“patients who experienced an RD event during a risk window for multiple study exposures of interest (ie multiple different types of antibiotics); these patients were excluded because a definite association with a single study exposure could not be assessed.”***

**The real world experience of RD occurrence during risk windows of potentially multiple antibiotics has therefore been removed from our analysis, to reach a more unbiased estimate.**

**This consideration was also added in the Discussion section, line 354-357.**
